# Serial determination of cyclic citrullinated peptide autoantibodies predicted five-year radiological outcomes in a prospective cohort of patients with early rheumatoid arthritis

**DOI:** 10.1186/ar1896

**Published:** 2006-01-26

**Authors:** Olivier Meyer, Pascale Nicaise-Roland, Marie dos Santos, Colette Labarre, Maxime Dougados, Philippe Goupille, Alain Cantagrel, Jean Sibilia, Bernard Combe

**Affiliations:** 1 Rheumatology Unit, Assistance Publique Hôpitaux de Paris, Bichat University Hospital, 75018 Paris, France; 2 Biological Immunology Department, Assistance Publique Hôpitaux de Paris, Bichat University Hospital, 75018 Paris, France; 3 Rheumatology Unit B, Assistance Publique Hôpitaux de Paris, Cochin University Hospital, 75014 Paris, France; 4 Rheumatology Unit, Trousseau University Hospital, 37044 Tours Cedex 1, France; 5 Rheumatology Unit, Rangueil University Hospital, 31043 Toulouse Cedex 4, France; 6 Rheumatology Unit, Hautepierre University Hospital, 67098 Strasbourg Cedex, France; 7 Rheumatology Unit, Lapeyronie University Hospital, 34296 Montpellier Cedex 5, France

## Abstract

The objective of this study was to evaluate the potential of serially determined anti-cyclic citrullinated peptide (CCP) antibodies for predicting structural joint damage in patients with early rheumatoid arthritis (RA), compared to a single baseline determination. Ninety-nine RA patients with disease durations of less than one year and no history of disease-modifying antirheumatic drug therapy were followed prospectively for at least five years. Anti-CCP2 concentrations were measured using a second-generation ELISA. Sharp scores as modified by van der Heijde were determined on hand and foot radiographs. Anti-CCP2 antibodies were detected in 55.5% of patients at baseline and 63.6% at any time during the first three years. Presence of anti-CCP2 at any time during the first three years was associated with radiographic damage at baseline (odds ratio (OR), 3.66; 95% confidence interval (95% CI) 0.99–13.54) and with five year progression of the total Sharp score (OR, 3.17; 95% CI, 1.3–7.7), erosion score (OR, 5.3; 95% CI, 1.4–19.2) and joint space narrowing score (OR, 2.8; 95% CI, 1.15–6.8). The presence of anti-CCP2 or IgM RF at baseline did not predict these outcomes. Patients with negative anti-CCP2 tests throughout follow-up had less radiographic progression than patients with increasing anti-CCP2 concentrations; they did not differ from patients with decreasing anti-CCP2 antibody levels. HLADRB1* typing showed that progression of the mean modified Sharp score was not correlated with the presence of the shared epitope alleles. In conclusion, serially determined anti-CCP2 antibodies during the first three years of follow-up performs better than baseline determination for predicting radiographic progression in patients with early RA.

## Introduction

Autoantibodies to citrullinated cyclic peptides (CCPs) were recently described as useful diagnostic markers for rheumatoid arthritis (RA) [1]. Studies that used the first-generation ELISA (CCP1) suggested that the presence of anti-CCPs might predict erosive disease in populations with early RA [2-7]. Similar results were obtained recently with the second-generation ELISA (CCP2) [8-10]. However, not all patients with anti-CCPs go on to experience erosive disease. Anti-CCP2 is associated with erosions and radiographic progression, but most of the odds ratios (ORs) reported to date are only modestly elevated, in the 2.5 to 3.5 range. Models combining several parameters have been built in an attempt to identify patients at high risk for severe disease progression. C-reactive protein combined with anti-CCP was the only significant predictor of joint destruction in the hands and feet after 10 years in a cohort of 176 patients with early RA at enrollment [10]. The HLA DR4 shared epitope combined with anti-CCP2 was the best combination for predicting severe disease progression in a study of 268 patients with early RA [9]. Thus, anti-CCP2 is emerging as a key tool for predicting joint damage in patients with early RA.

We investigated whether the predictive value of anti-CCP2 for radiographic joint damage in RA could be improved by repeating the assays over time. To this end, we compared baseline anti-CCP2 versus serial anti-CCP2 assays throughout the first three years. Sensitivity and the OR for predicting joint damage were determined for each strategy.

## Materials and methods

### Patients

Ninety-nine patients (72 female and 27 male) who met at least four 1987 American College of Rheumatology criteria for RA [11] and had disease duration of less than one year were followed prospectively for at least five years. Patients were part of an early-RA cohort (called the Montpellier-Cochin-Tours/Toulouse (Mo-Co-To) cohort) of 191 patients reported previously [12]. At enrollment, none of the patients had experience with disease-modifying antirheumatic drugs (DMARDs). During the first 3 years of follow-up, all but 3 patients received methotrexate alone (7.5 to 15 mg/week; *n* = 38), sulfasalazine alone (2.5 g/day; *n* = 31), or both drugs in combination (*n* = 27). Oral corticosteroids (prednisolone, 5 to 15 mg/day) were received by 33 patients. No patients were treated with biological agents.

The study protocol was approved by the appropriate ethics committee. All the patients signed an informed consent document.

### Methods

Sera obtained at baseline and after one and three years were stored at -20°C until use. Anti-CCP2 was assayed using a commercial ELISA kit (Immunoscan RA mark 2, Eurodiagnostica, Arnhem, The Netherlands) according to the manufacturer's instructions. Antibody concentrations are given as a continuous variable from 25 U/ml to >15,200 U/ml). The upper limit of normal (cutoff) was 50 U/ml. In addition, immunoglobulin M rheumatoid factors (IgM RFs) were assayed using an in-house ELISA and considered positive when ≥ 20 IU/ml. Patients were classified according to the cutoff value of the serological tests as IgM RF positive or negative and anti-CCP2 positive or negative, at baseline and at later time points. Patients with anti-CCP2 antibodies (*n* = 63) were further classified into three groups according to the anti-CCP2 concentration change between baseline and month 36, as follows: no change, defined as a positive value (>50 U/ml) with a smaller than 30% variation from baseline (*n* = 12); decrease, defined as a greater than 30% drop from baseline (*n* = 32), including patients with conversion from positive to negative by the end of the follow-up; and increase, defined as a greater than 30% elevation from baseline (*n* = 19) or conversion from negative (<50 U/ml) to positive. The 36 other patients had no anti-CCP2 antibodies at any of the study time points.

Radiographic measurements at the hands and feet were taken at baseline and after three and five years. Radiographs were evaluated by two independent observers who were unaware of the patient data. The observers used the Sharp method as modified by van der Heijde [13]. For each patient, an erosion score, a joint space narrowing score, and a total damage score calculated as the sum of the first two scores were determined for the hands and feet. At baseline, 19 patients (20%) had significant structural damage (total Sharp score higher than 5.5).

To determine a cutoff value above which score changes indicated individual radiographic progression unrelated to measurement error (that is, the smallest detectable difference), we calculated the mean of the differences between two measurements, as described previously [14]. As recommended by OMERACT [15], we defined radiographic progression as a radiographic score change greater than the upper boundary of the 95% confidence interval (95% CI) of the relevant difference. After five years, this upper boundary was 4.1, 3.2 and 5.5 for the erosion score, narrowing score and total score, respectively. Using this definition of radiological progression, after five years 50 patients had no radiographic progression and 49 had progression of one or more radiographic scores (total score, *n* = 47; erosion score, *n* = 25; and narrowing score, *n* = 45).

HLA DR typing and subtyping were performed by PCR using specific primers and hybridization with sequence-specific oligonucleotides. DRB1 alleles *0101, *0102, *0401, *0404, *0405 are prevalent shared epitope (SE) alleles found in our RA population. The prevalence of the shared epitope in the control French population is 37.1% [16].

### Statistical analysis

The chi-square test was used to examine concordance between one (baseline) and multiple anti-CCP2 determinations and between IgM RFs at baseline and after three years. We evaluated the effectiveness of anti-CCP2 and IgM RFs at baseline and over time for predicting radiographic progression after five years. Patients were separated into two groups, with and without radiographic progression. The OR with the 95% CI for significant radiographic progression was calculated, as well as the sensitivity and specificity of anti-CCP2 for predicting radiographic progression after five years. The analysis was then repeated after stratification of patients according to their anti-CCP2 status during follow-up, as follows: persistent negative anti-CCP2 test; unchanged anti-CCP2 concentration; increased anti-CCP2 concentration (with a switch from negative (<50 U/ml) to positive in 7 of 19 patients); and decreased anti-CCP2 concentration (with a switch from a positive to a negative (<50 U/ml) test in 8 of 32 patients).

Data were also analyzed with the anti-CCP2 concentration and the radiographic Sharp score as continuous variables. The nonparametric Spearman test was used to evaluate correlations linking the progression of the radiographic Sharp scores to the baseline anti-CCP2 concentration and to the mean serial anti-CCP2 concentration computed as ∑(M0+M12+M36)/3, where M0 is month 0 (baseline), M12 is month 12 and M36 is month 36.

We tested the hypothesis that patients with persistent or increasing anti-CCP2 concentrations were more likely to show radiographic progression than patients with persistently negative anti-CCP2 tests. We compared more than one anti-CCP2 determination to one determination at baseline for predicting radiographic progression after five years. The ORs with their 95% CIs were computed. We used the Mann-Whitney test to compare the erosion, narrowing and total scores across patients categorized based on anti-CCP2 over time and to compare anti-CCP2 concentrations in patients with or without SE alleles. Finally, the chi-square test was used to evaluate the presence of anti-CCP2 according to the presence of one or more SE alleles. Differences were considered statistically significant when *P *was smaller than 0.05 and when the 95% CI did not include 1.

## Results

Clinical features and laboratory test results in the 99 patients with early RA (Table 1) were not different from those in the entire Mo-Co-To cohort, which have been published elsewhere [12].

**Figure 1:**
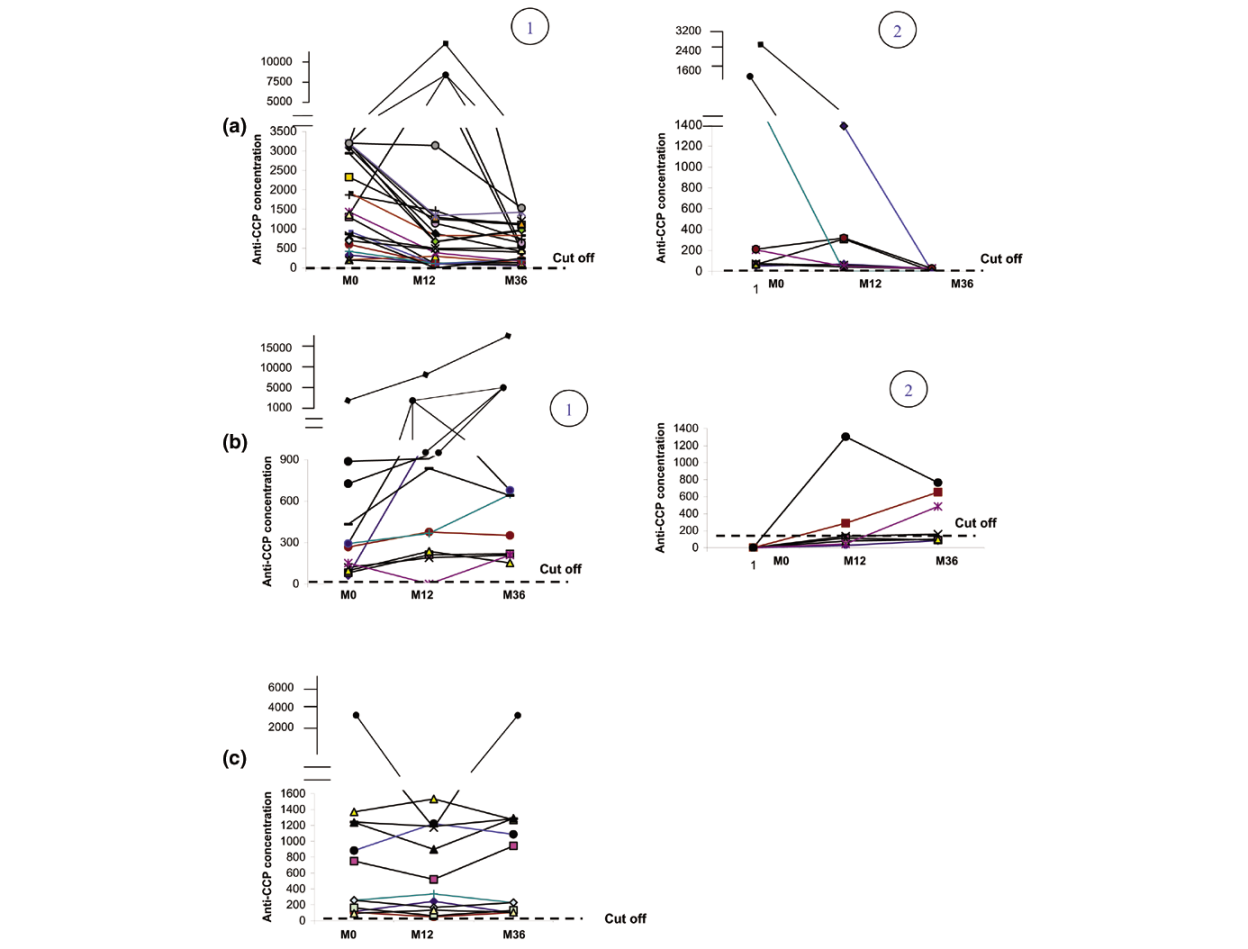
Anti-CCP2 concentrations at baseline (month 0 (M0)), 1 year (M12) and 3 years (M36) of follow-up according to **(a) **decreasing concentrations (panel 1 and 2), **(b) **increasing concentrations (panel 1 and 2) or **(c) **steady concentrations. Patients with transition from positive to negative and negative to positive are shown on panel 2 in (a, b).

Sensitivity for RA of a positive anti-CCP2 test in our population of 99 patients with early RA was 55.5% at baseline and 63.6% at any time during the first three years of follow-up. In seven (7%) patients, anti-CCP2 was negative at baseline but converted to positive within the first three years, whereas in 8 (8%) patients anti-CCP2 was positive at baseline but converted to negative within the first three years (Figure 1a, b, panel 2). Among 19 patients with increasing anti-CCP2 concentrations during the first three years of follow-up, seven were treated with methotrexate (MTX; 37%), seven with sulfasalazine (SSZ; 37%), and five with both drugs in combination (MTX + SSZ; 26%). Of these 19 patients, seven had no anti-CCP antibodies at baseline and converted to positive during follow-up (2 treated with MTX, 3 with SSZ, and 2 with MTX + SSZ). Among 32 patients with decreasing anti-CCP2 concentrations, 14 were treated with MTX (43.75%), 10 with SSZ (31.25%), 6 with MTX + SSZ, and 1 with hydroxychloroquine; 1 patient received no DMARDs. Of the 32 patients who were anti-CCP2 positive at baseline, 8 converted to negative by the end of the first three years of follow-up (5 treated with MTX, 1 with SSZ, 1 with MTX + SSZ, and 1 with hydroxychloroquine). None of the differences in DMARD regimens across these subgroups was statistically significant. Serum IgM RF was detected in 73.7% of patients at baseline and in 83.8% at some time during the first three years. IgM RF titer status changed within the first three years in 45 patients, of whom 34 converted from positive to negative and 11 from negative to positive (data not shown).

Significant structural damage was present at baseline in 20 patients and after five years in 59 patients (total Sharp score). Presence of anti-CCP2 at the first determination was not significantly associated with radiographic damage at baseline (13/55 (23.6%) patients with anti-CCP and 7/44 (15.2%) patients without anti-CCP; OR, 1.63; 95% CI, 0.59–4.54; *P *= not significant).

Presence of anti-CCP2 at any time during the first three years was associated with radiographic damage at the hands and feet at baseline (17/63 (27%) patients with anti-CCP and 3/36 (8.3%) patients without anti-CCP; OR, 3.66; 95% CI, 0.99–13.54; *P *= 0.063).

To investigate the value of a positive anti-CCP2 test for predicting radiographic progression, we computed the ORs for radiographic progression after five years with the serial anti-CCP2 strategy (Table 2) and we compared the results to those obtained with anti-CCP2 determination at baseline only. Table 2 reports the ORs for radiographic progression according to serial IgM RF values and to baseline IgM RF status. The 95% CI values showed that presence of anti-CCP2 at any time during the first three years significantly predicted erosions (*P *= 0.007), joint space narrowing (*P *= 0.03), and total score deterioration (*P *= 0.01), whereas the presence of anti-CCP2 detected by a single determination, at baseline, predicted none of these outcomes.

Figure 1 summarizes the anti-CCP2 antibody level variations among patients with increasing concentrations and patients with decreasing concentrations between baseline and month 36. The median antibody level increase (Δ M36 minus M0; 207 U/ml; range, 57 to 1,190) did not differ significantly from the median antibody level decrease (Δ M0 minus M36; 1003 U/ml; range, 27 to 3,200).

Erosions were noted after five years in 35% (22/63) of patients with positive anti-CCP2 at any time during the first three years and in 8.3% (3/36) of patients with negative anti-CCP2 throughout the first three years (two patients had a transiently and weakly positive anti-CCP2 test at 12 months) (Figure 1). Among patients with and without anti-CCP2 during the first three years, 54% (35/65) and 29% (10/34) had joint space narrowing, respectively, and 57% (37/65) and 29% (10/34) experienced total score deterioration, respectively. Figure 3 reports the mean (± standard deviation) changes in the erosion score, narrowing score and total score after five years according to anti-CCP2 variations. The mean erosion score and total score were significantly higher in patients with increasing anti-CCP2, compared to those with decreasing antibody levels. Patients with negative anti-CCP2 tests throughout follow-up had less structural damage (erosions, narrowing and total score) than did patients with increasing anti-CCP2 concentrations. Finally, regarding structural deterioration, patients with stable anti-CCP2 concentrations did not differ from those with increasing or decreasing anti-CCP2 titers.

**Figure 2:**
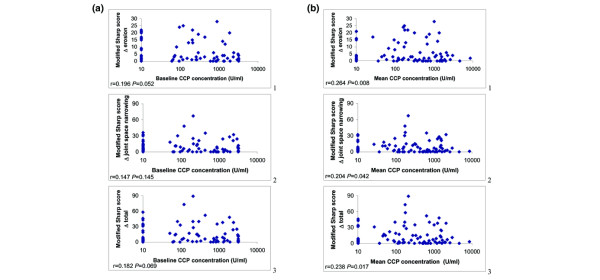
Correlation between **(a) **anti-cyclic citrullinated peptide (CCP) concentrations at baseline (month 0 (M0); panels 1 to 3) or **(b) **the mean serial anti-CCP concentrations (M0 + M12 + M36)/3; panels 1 to 3) and progression of the modified Sharp scores (Δ erosion, Δ joint space narrowing and Δ total score).

**Figure 3:**
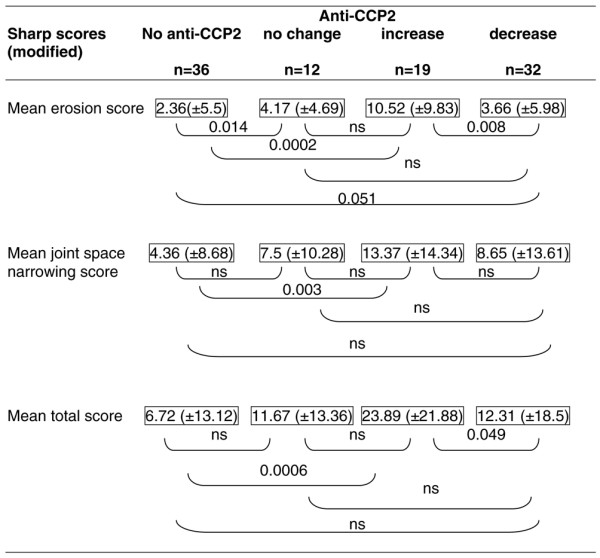
Progression of radiographic Sharp scores (mean ± SD) after five years in patients with or without anti-CCP2 antibodies, according to the change in anti-CCP2 concentrations between baseline and three years.

We evaluated correlations between the baseline anti-CCP2 antibody level, or the mean of the three anti-CCP2 antibody levels, and the Sharp score changes after five years (erosion score, narrowing score and total score) in the 99 patients. Correlation coefficients for the mean of the three anti-CCP2 concentrations are reported in Table 3. All three radiographic scores were significantly correlated with the mean serial anti-CCP2 concentrations, whereas the correlations with the baseline anti-CCP2 concentration fell slightly short of significance (Figure 2).

One or two SE alleles (*0401, *0404, *0405, *0101, or *0102) were found in 68% of patients. This proportion was higher in patients with than in patients without anti-CCP2 (61% and 50%, respectively), although the difference was not statistically significant. The mean serial anti-CCP2 concentrations were compared in patients with and without the SE alleles. No significant differences were found between these two subgroups (705 ± 1,408 U/ml versus 450 ± 632 U/ml). The 15 patients with the *0404 allele had a significantly higher mean serial anti-CCP2 concentration (1,163 ± 2,247 U/ml) compared to the other patients (491 ± 776 U/ml) (*P *= 0.036), whereas no significant difference was found for the baseline anti-CCP2 concentration. We compared five-year total Sharp scores according to the presence or absence of anti-CCP2 during the first 3 years in patients with or without SE alleles. As shown in Table 4, the mean modified Sharp scores were significantly higher in patients with a positive anti-CCP2 test at any time during the first three years than in patients without anti-CCP2 throughout the first three years, in both the subgroup with and the subgroup without SE alleles.

## Discussion

The course and outcome of RA vary according to several parameters, including disease activity, functional status, constitutional symptoms and joint damage. In this study, we focused on the value of anti-CCP2 autoantibodies for predicting joint damage. Many studies have established that the presence of anti-CCP strongly predicts progression to RA in patients with early arthritis [1]. Anti-CCP antibodies appear early and may antedate symptom development [17]. However, little is known about the time-course of anti-CCP during the early phase of RA [18] in the absence of anti-tumor necrosis factor-α therapy. Furthermore, the presence of anti-CCP in early RA may predict erosive disease [3-5,8,10,18-23]. We showed [5] that anti-CCP2 was superior over IgM RF for predicting joint damage progression over three or five years. This finding does not imply that all RA patients with anti-CCP2 will experience rapidly progressive joint damage. In the present study, only 57% of patients with anti-CCP2 at any time during the first three years experienced significant joint damage progression within the first five years.

To improve knowledge of the value of anti-CCP2 for predicting radiographic joint damage, we compared one anti-CCP2 determination at baseline to the mean of three anti-CCP2 determinations, at baseline and after one and three years, respectively, in a cohort of patients with early RA. Follow-up radiographs were taken after five years. Of the 191 patients in the Mo-Co-To early-RA cohort [12], 99 had at least three anti-CCP2 determinations at the required time points, as well as radiographs at baseline and five years later. These 99 patients were included in the present study. Joint damage progression was defined as the smallest detectable difference, which can serve as the minimal clinically important difference, on hand and foot radiographs [15]. Structural damage at baseline was associated with anti-CCP2 at any time during the first three years but not with anti-CCP2 at baseline only. These data contrast with our previous finding that anti-CCP at baseline predicted structural damage during the next five years [5]. However, this apparent discrepancy may be ascribable to the small number of patients included in the present study: only 99 of the 191 patients in the Mo-Co-To cohort were included, based on availability of anti-CCP2 titers after one or three years. The entire cohort of 191 patients [12] and the 99 patients in the present study did not differ significantly in terms of sex ratio, percentage of patients with anti-CCP or IgM RF at baseline, HLADRB1 alleles, or other parameters reflecting disease activity. The statistical power afforded by the sample size of 99 patients may have been inadequate to detect a significant association.

The main message from our data is that serial anti-CCP2 determination is better than a single baseline determination for predicting five year progression of erosions, joint space narrowing, and total Sharp score as modified by van der Heidje. The ORs were 5.28 for erosions, 3.17 for the total score, and 2.8 for joint space narrowing. In contrast, a positive IgM RF test at baseline or at any time during the first three years failed to predict progression of radiographic structural damage, in contradiction to previous reports [3,10,18,22]. This discrepancy may be ascribable to the small proportion (26%) of patients in our study who were negative for IgM RF at baseline, which decreased the likelihood of finding a statistically significant difference. IgM RF also failed to predict radiographic progression in our previous study of 133 of the 191 Mo-Co-To cohort patients [5], in contrast to results with the entire Mo-Co-To cohort [12]. Statistically significant correlations were found between the mean serial anti-CCP2 concentration and progression of the erosion score (r = 0.264), joint space narrowing score (r = 0.204), and total score (r = 0.238) in the 99 patients. In contrast, the baseline anti-CCP2 concentration was not significantly correlated with radiographic progression. This finding agrees with recent data from Boire and colleagues [24] drawing attention to the prognostic significance of antiSa and other citrullinated antigen-antibody systems that are highly specific for RA and that better predict early structural damage than does the baseline anti-CCP2 concentration.

We sought to improve the use of anti-CCP2 as a predictive tool by dividing the patients into three categories based on anti-CCP2 concentration changes during the first three years of follow-up. An increase in the anti-CCP2 antibody concentration was seen in 19 patients (30% of the patients with anti-CCP2) and a decrease in 32 patients (51%). The few published reports of anti-CCP2 antibody level elevation during treatment with nonbiological DMARDs [25] include patients in the Swedish TIRA study [18] and individual Japanese patients treated with various DMARDs [26]. All patients (except three) from our Mo-Co-To cohort were treated with methotrexate, sulfasalazine, or both, and no difference in DMARD regimen was seen between patients with decreasing anti-CCP2 concentrations and those with increasing anti-CCP2 concentrations. Mean erosion, joint space narrowing and total scores were significantly higher in patients with increasing anti-CCP2 concentrations than in those without anti-CCP2. The mean erosion and total scores were also significantly higher in patients with increasing concentrations than in those with decreasing concentrations.

The anti-CCP2 concentration during the first three years of the disease was independent from DRB1 HLA status, most notably regarding the presence of SE alleles; the only exception was DRB1* 0404, which was associated with higher levels of anti-CCP2 compared to the other patients. Our data suggest that patients with *0404 DRB1 may be more prone to develop high anti-CCP antibody levels during the first three years. These data are only partly in accordance with previous studies of cohorts from northern Europe [9,23,27-29], in which anti-CCP (in any titer) was associated with the whole SE DRB1*04 or with the *01 or *10 alleles. However, our purpose was not to correlate the presence or absence of anti-CCP with the DRB1* alleles carrying the SE but, instead, to determine whether the anti-CCP concentration was related to specific SE DRB1* alleles. Our finding of an association with DRB1* 0404 is in accordance with another recent French study [30].

## Conclusion

Taken together, these data indicate that anti-CCP2 concentrations determined serially during the first three years of RA might be good predictors of subsequent radiographic progression. Among anti-CCP2-related parameters, an increase in anti-CCP2 antibody levels during the first three years is correlated with radiographic progression within the first five years.

## Abbreviations

CCP = cyclic citrullinated peptide; CCP1 = first-generation CCP test; CCP2 = second-generation CCP test; CI = confidence interval; DMARD = disease-modifying anti rheumatic drug; ELISA = enzyme linked immunosorbent assay; IgM M0 = month 0 (baseline); M12 = month 12; M36 = month 36; Mo-Co-To = Montpellier-Cochin-Tours/Toulouse cohort; MTX = methotrexate; OR = odds ratio; RA = rheumatoid arthritis; RF = immunoglobulin M rheumatoid factor; SE = shared epitope; SSZ = sulfasalazine.

## Competing interests

The authors declare that they have no competing interests.

## Authors' contributions

OM was a main investigator, designed the investigation, and was the main contributor to the preparation of the manuscript. PNR participated in the discussion, was responsible for assay of the anti-CCP antibodies, and contributed to the preparation of the manuscript. MDS participated in the analysis of the anti-CCP antibodies. CL is responsible for the Immunology Unit and participated in the discussion. MD was a main clinical investigator and made a major contribution to the preparation of the manuscript. PhG was a main clinical investigator and contributed to the preparation of the manuscript. AC was a main clinical investigator and contributed to the preparation of the manuscript. JS was a main clinical investigator, contributed to the preparation of the manuscript, and was responsible for the analysis of the rheumatoid factors. BC was a main clinical investigator, contributed to the preparation of the manuscript, and was involved in all aspects of the study.

## Figures and Tables

**Table 1:** Baseline characteristics in 99 patients with early rheumatoid arthritis

Female (%)	72
Mean age (years)	50.7
Mean disease duration (months)	4.3
Pain (100 mm VAS)	54.4
Morning stiffness (minutes)	84
Ritchie index	17.8
Tendon joint count	21.8
Swollen joint count	8.4
Nodules (%)	6
ESR (mm/1^st ^h)	38.4
CRP (mg/l)	32
IgM RF positive (%)	71
DAS 44	4.1
HLA DRB1*04 (%)^a^	53
HLA DRB1*01 (%)^b^	15.5

^a^DRB1*04 includes DRB1*0401, *0404, *0405, and *0408. ^b^DRB1*01 includes DRB1*0101 and *0102. CRP, C-reactive protein; DAS, disease activity score; ESR, erythrocyte sedimentation rate; IgM RF, immunoglobulin M rheumatoid factor; VAS, visual analog scale for pain.

**Table 2:** Anti-CCP and IgM RF as predictors of radiographic joint destruction after 5 years

	Odds ratios (95% confidence intervals)		
	Erosions	Joint space narrowing	Total

Anti-CCP2 (baseline)	1.91 (0.73–4.96)	1.64 (0.73–3.68)	1.90 (0.85–4.27)
Anti-CCP2 (first 3 years)	5.28 (1.45–19.2)^a^	2.8 (1.15–6.78)^b^	3.17 (1.30–7.69)^c^
IgM RF (baseline)	0.45 (0.17–1.17)	0.57 (0.23–1.39)	0.67 (0.28–1.65)
IgM RF (first 3 years)	0.70 (0.21–2.25)	1.03 (0.35–3.0)	0.88 (0.30–2.58)

^a^*P *= 0.007; ^b^*P *= 0.03; ^c^*P *= 0.01. IgM RF, immunoglobulin M rheumatoid factor.

**Table 3:** Correlation between worsening of radiographic Sharp score and mean serial anti-CCP2 concentration or baseline anti-CCP2 concentration

Sharp score	Mean anti-CCP2 concentration Δ(M0 + M12 + M36)/3		Anti-CCP2 concentration baseline (M0)	
	r (95% CI)	Pa	r (95% CI)	Pa

Erosions	0.264 (0.06–0.44)	0.008	0.196 (-0.007–0.383)	0.052
Joint space narrowing	0.204 (0.002–0.392)	0.042	0.147 (-0.057–0.340)	0.145
Total	0.238 (0.037–0.421)	0.017	0.182 (-0.02–0.372)	0.069

The serial anti-CCP2 concentration is the mean of three determinations. aSpearman test. CI, confidence interval; M0, month 0; M12, month 12; M36, month 36; r, correlation.

**Table 4:** Mean radiographic Sharp scores after 5 years in patients with or without anti-CCP2 and shared epitope alleles

	Anti-CCP2		*P* value
	Anti-CCP2		*P* value
Yes	17.2 ± 21.1	9.7 ± 17.3	0.0021

	NS	NS	
No	14.4 ± 16.3	2.7 ± 3.7	0.031

NS, not significant; SE, shared epitope.
